# The relationship between IgG and IgM levels and severity of symptoms in COVID-19 patients confirmed by rapid antigen test

**DOI:** 10.25122/jml-2021-0194

**Published:** 2021

**Authors:** Jasim Saeed Salman AL-Ibraheemi, Abdul-Sattar AL-Saeedi

**Affiliations:** 1. College of Medicine, University of Al-Ameed, Karbala, Iraq; 2. Basic and Medical Sciences Branch, College of Nursing, University of Al-Ameed, Karbala, Iraq

**Keywords:** antigen rapid test, SARS CoV-2, IgG, IgM, clinical symptoms

## Abstract

This study aims to measure immunoglobulin G (IgG) and immunoglobulin M (IgM) response after detection of Severe Acute Respiratory Syndrome coronavirus (SARS CoV-2) antigens in coronavirus disease 2019 (COVID-19) patients concerning the severity of symptoms. SARS CoV-2 antigen was confirmed by rapid antigen test, and IgG and IgM were confirmed by VIDAS® SARS-COV-2 IgM and VIDAS® SARS-CoV-2 IgG automated qualitative assays used to rapidly detect antibodies 20–30 days after detection. The serological assay for detecting SARS-CoV-2 IgM and IgG antibodies shows a positive correlation for all patients detected with SARS-CoV-2 antigen with sensitivity 100% with differences in antibodies levels between patients regarding age and significantly related clinical symptoms with p-value 0.013 <0.05. The appearance of clinical symptoms was not significantly related to IgG levels at a p-value of 0.4 >0.05. However, the appearance of clinical symptoms was significantly related to IgM levels at a p-value of 0.002 <0.05. Antigen-dependent rapid tests can be used to detect SARS-CoV-2 in an early stage of infection with high sensitivity and specificity. Moreover, this study shows the age groups 21–30 and 31–40 have a better response to SARS-CoV-2 infection.

## Introduction

The coronavirus disease 2019 (COVID-19) is a relatively new virus, also known as Severe Acute Respiratory Syndrome coronavirus-2 (SARS-CoV-2), which has spread globally since late 2019 [1–5]. The S glycoprotein from the surface of the virus is responsible for the SARS-CoV-2 bond and enters host cells through its binding to the angiotensin-converting enzyme 2 (ACE-2) receptor [6]. Virus-specific neutralizing antibodies (NAbs) have a major role in decreasing viral replication and increasing clearance from viruses [6]. Neutralizing antibodies mainly bind and block the receptor-binding domain (RBD) on the surface of the SARS-CoV-2 S protein [7–9]. The humoral immune (antibody) response remains for at least three weeks and, in some cases, even longer [10]. Other studies of severe acute respiratory syndrome coronavirus (SARS-CoV) and Middle East respiratory syndrome coronavirus (MERS-CoV) indicate that the highly immunogenic antigens are the S and N viral proteins, and the progress of serological tests such as enzyme-linked immunosorbent assay and magnetic Chemiluminescence enzyme immunoassay for SARS-CoV-2 immunoglobulin (Ig) G or IgM antibodies has focused on these proteins[11]. The serologic assay is the primary focus in identifying the presence of antibodies response against the SARS-CoV-2 antigen, for either herd immunity and monitoring seroprevalence (epidemiological purposes) or for complementing nucleic acid amplification tests (NAATs) in specific circumstances [12–14]. Until now, the number of antigen-based diagnostic tests is lower than those available for antibody detection. Among the four structural proteins (S, E, M, and N protein) of SARS-CoV-2, S and N proteins are the main immunogenic protein [15–17]. Complementary to molecular genetics assays are the serological rapid antigen tests that give detection of viral surface antigens [18]. These tests are dependent on certain monoclonal antibodies to produce a mechanism for the bind of viral antigens from an analytical sample. These assays are not limited to a particular format, such as involving a colorimetric enzyme immunoassay for SARS-CoV in 2004 [19]. In this study, patients diagnosed with COVID-19 via rapid antigen test were tested for their immunoglobulin G (IgG) and immunoglobulin M(IgM) levels based on the serological assay 20–30 days after initial detection. The antibody levels were analyzed according to clinical manifestation (whether symptomatic, pauci-symptomatic, or asymptomatic), age, and sex.

## Material and Methods

### Study design and participants

This study was promoted by the Alkafeel Super Specialty Hospital and the University of Al-Ameed Karbala Iraq during the COVID- 19 pandemic. 30 COVID-19 patients, confirmed by rapid antigen test, were screened for SARS-CoV-2 IgG and IgM levels at the microbiology lab of Alkafeel Hospital (Vito) between January 4 and April 1, 2021.

### Data Collection

The demographics, clinical signs, symptom profile, and outcome data were obtained from standardized case report software in the hospital. In some cases, follow-up calls were made for more details.

### Antigen assay

#### Test Preparation

First, all kit components should reach a temperature between 15–30°C prior to testing. Following this, the test device is removed from the foil pouch and placed on a flat and clean surface. Next, the extraction tube should be full of buffer fluid to fill-line (300 μl) [20].

#### Specimen Collection & Extraction

Specimen Collection and Extraction were done according to the manufacturer’s instruction in the PanbioCOVID-19 Ag Rapid Test Device handbook. The patient’s head was tilted back 70 degrees, and the swab was gently rotated. The swab was inserted less than one inch (about 2 cm) into the nostril (until resistance was met at the turbinates). The swab was rotated five times against the nasal wall then slowly removed from the nostril. The same swab was used to repeat the collection procedure with the second nostril. The swab tip was swirled in the buffer fluid inside the extraction tube, pushed into the wall of the extraction tube at least five times, and squeezed out the swab. The swab was broken at the breakpoint, and the cap of the extraction tube was closed [20].

#### Reaction with Test Device

**Figure 1.**
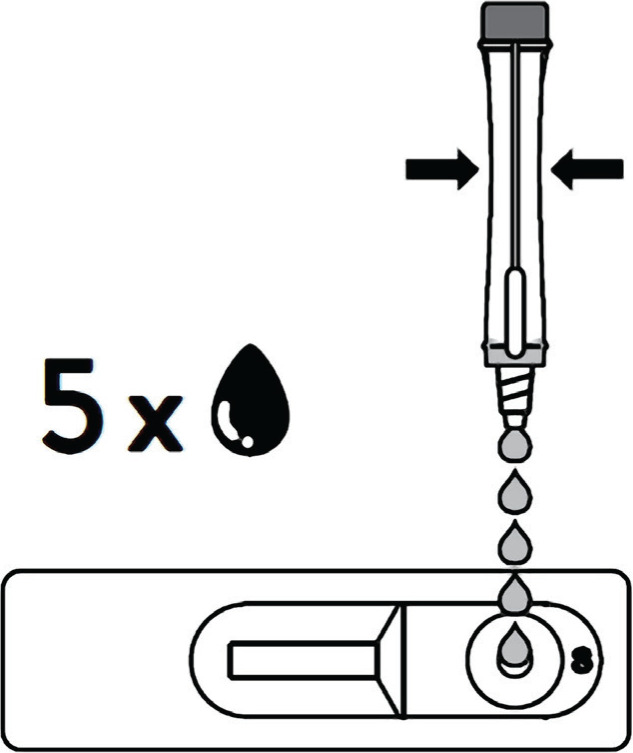
5 drops of extracted specimens were dispensed vertically into the specimen well (S) [20].

The dropping nozzle cap should open at the bottom of the extraction tube, and then 5 drops of extracted specimens were dispensed vertically into the specimen well (S) on the device, as shown in Figure 1. Following that, we disposed of the extraction. After 15 minutes, the result appears on the disposal of the device [20].

### IgG and IgM assay

The tests were performed according to the manufacturer’s instruction in VIDAS® SARS-COV-2 IgM and VIDAS® SARS-COV-2 IgG automated qualitative assays that rapidly detect antibodies.

### Determining Symptom Classification

Pauci-symptomatic patients developed symptoms such as cough, fever, fatigue, and sore throat. Patients with mild symptoms were defined as patients with respiratory symptoms, fever, and mild pneumonia. Patients with severe symptoms were defined as patients who suffered from difficulty breathing, abnormal blood gas analysis, hypoxia, and severe pneumonia. Finally, patients with respiratory failure (severe acute respiratory syndrome) were named critical patients [21]. Asymptomatic infections were defined as positive antigen test results but without clinical symptoms in the past 14 days [22].

### Statistical analysis

The data calculations were performed using Microsoft Excel 2016. Statistical analyses were performed using SPSS software, version 26. Graphs were created in SPSS software, version 26. IgG, IgM, age, and clinical symptoms were analyzed using chi-Square Tests. The significance of the statistical value was determined at p <0.05.

## Results

The nasopharyngeal swab obtained between January 4, 2021, and April 1, 2021, was positive for SARS-CoV-2 on rapid antigen testing for 30 patients. After 20–30 days, the serological assay for detecting SARS-CoV-2 IgM and IgG antibodies showed a positive value for all patients detected with SARS-CoV-2 antigen with sensitivity 100% with differences in antibodies levels between patients. Table 1 summarizes the demographic and clinical characteristics of the 30 patients tested, of which 8 were female (20%), and 22 (73%) were male. From the female demography, 3 were pauci-symptomatic, and 5 were symptomatic, whereas, for male participants, 2 were symptomatic, 9 were pauci-symptomatic, and 11 were symptomatic.

**Table 1.** Demographic and clinical characteristics of COVID-19 patients.

**Characteristics**	**Age groups**	**Frequency**
**Age**	<10	1 (3.3%)
	21–30	10 (33.3%)
	31–40	10 (33.3%)
	41–50	8 (26.7%)
	61+	1 (3.3%)
	Total	30 (100%)
**Gender**	Males 22 (73 %)	
	Female 8 (26%)	
**Clinical status**	Asymptomatic 2 (6.66%)	
	Pauci-symptomatic 14 (46.66%)	
	Symptomatic 14 (46.66%)	

**Figure 2.**
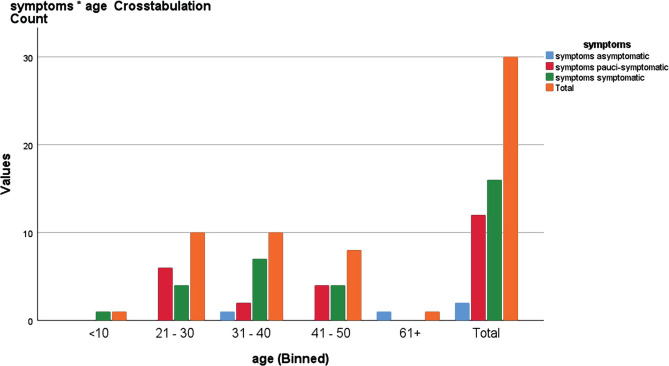
Relationship between age and symptoms as determined by Chi-square test.

Patients were further classified according to age group. There was only one symptomatic patient in the less than 10 years old age group. The 21–30 age group had 6 pauci-symptomatic patients and 4 symptomatic patients, while for the 31–40 age group, 1 was asymptomatic, 2 pauci-symptomatic, and 7 symptomatic. In the 41–50 age group, 4 were pauci-symptomatic and 4 symptomatic. Finally, only one was asymptomatic in the 60+ age group, as shown in Figure 2. In this study, most SARS-CoV-19 infections were in the 21–30 and 31–40 age group, and the patients’ age was significantly related to clinical symptoms with a p-value of 0.013 <0.05, as shown in Table 2.

**Table 2.** Relationship between age and symptoms as determined by Chi-square test.

**Chi-Square Tests**	**Value**	df *	**Asymptotic Significance (2-sided)**
**Pearson Chi-Square**	19.312 ^a^	8	.013
**Likelihood Ratio**	12.352	8	.136
**Linear-by-Linear Association**	1.810	1	.178
**N of Valid Cases**	30		

a .13 cells (86.7%) have an expected count of less than 5. The minimum expected count is .07. * – Degrees of Freedom.

**Figure 3.**
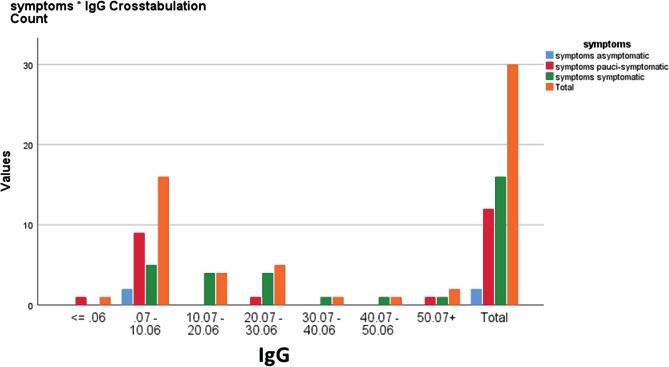
Relationship between IgG levels and symptoms as determined by Chi-square test.

The time interval between Antigen detection and the IgG and IgM testing ranged from 20 to 30 days. Regarding the antibody concentration IgG with symptoms for ≤0.06, only one was pauci-symptomatic, and for 0.07–18.76 ranges, two were asymptomatic, 9 were pauci-symptomatic, 9 were symptomatic, while for the 18.77–37.46 range, only one was pauci-symptomatic and 5 were symptomatic. Finally, for the ≥37.47 range, one was pauci-symptomatic, and 2 were symptomatic, as shown in Figure 3. The difference in IgG antibody levels between asymptomatic, pauci-symptomatic cases and symptomatic cases found that the appearance of clinical symptoms was not significantly related to IgG levels with a p-value of 0.4 >0.05 as shown in Table 3.

**Table 3.** Relationship between IgG levels and symptoms as determined by Chi-square test.

**Chi-Square Tests**	**Value**	df *	**Asymptotic Significance (2-sided)**
**Pearson Chi-Square**	4.781 ^a^	6	.572
**Likelihood Ratio**	5.756	6	.451
**Linear-by-Linear Association**	2.526	1	.112
**N of Valid Cases**	30		

a .10 cells (83.3%) have an expected count of less than 5. The minimum expected count is .07. * – Degrees of Freedom.

**Figure 4.**
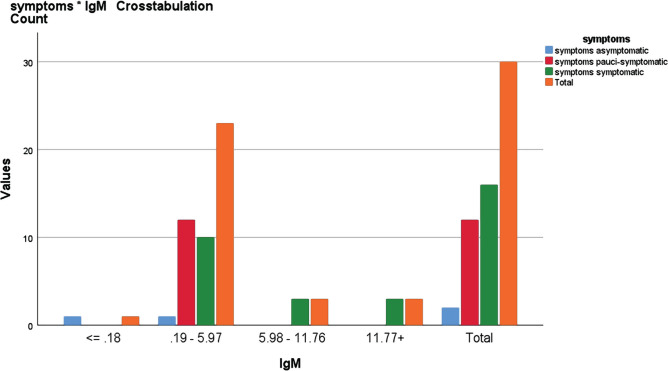
Relationship between IgG levels and symptoms as determined by Chi-square test.

In relation to IgM concentration with symptoms for the ≤0.18 range, only one was asymptomatic, but in the 0.19–5.97 ranges, one was asymptomatic, 12 were pauci-symptomatic, and 10 were symptomatic. For the 5.98–11.76 range, 3 were symptomatic, as shown in Figure 4. The difference in IgM antibody levels between asymptomatic, pauci-symptomatic cases and symptomatic cases found that the appearance of clinical symptoms was significantly related to IgM levels with a p-value of 0.002 <0.05, as shown in Table 4.

**Table 4.** Relationship between IgM levels and symptoms as determined by Chi-square test.

**Chi-Square Tests**	**Value**	df *	**Asymptotic Significance (2-sided)**
**Pearson Chi-Square**	20.707 ^a^	6	.002
**Likelihood Ratio**	14.395	6	.026
**Linear-by-Linear Association**	7.162	1	.007
**N of Valid Cases**	30		

a .10 cells (83.3%) have an expected count of less than 5. The minimum expected count is .07. * – Degrees of Freedom.

## Discussion

In the present study, a group of 30 COVID-19 patients positive for the SARS-CoV-2 antigen test was tested to detect IgG and IgM levels 20–30 days after antigen testing. In this study, after 20–30 days, the serological assay for detecting SARS-CoV-2 IgM and IgG antibodies shows positive values for all patients detected with SARS-CoV-2 antigen, achieving a sensitivity of 100% with differences in patients’ antibodies levels. In a previous study, antigen detection had given a high true positive rate and false-negative rate, which can be taken as an early diagnostic marker for SARS one day before clinical symptoms developed [23]. However, the antigen detection achieved a sensitivity of 100%, which greatly reduced the false positive rate of nucleic acid detection. Moreover, patients who present with three days of fever at the earliest can be identified by the rapid antigen test [18], which agrees with our findings. In this study, most SARS-COV-2 infections were detected in the age groups of 21–30 and 31–40 years old. The patients’ age is significantly related to clinical symptoms with a p-value of 0.013 <0.05. This evidence was highlighted in a study that identified that the rising COVID-19 epidemics in the US in 2020 grew among adults aged 20–49. In particular, adults aged 35 to 49 accounted for an estimated 72.2% (68.6 to 75.9%) of SARS-CoV-2 infections in the US with locations considered, whereas less than 5% originated from children aged 0 to 9, and less than 10% from teens aged 10 to 19 [24]. Another study showed that older subjects have significantly less close contact than younger subjects [25]. This indicates that older people may be more aware of maintaining social distance, wearing protective masks and gloves, and having good hygiene behavior. Testing antibodies against SARS-CoV-2 is rapid and sensitive for the auxiliary diagnosis of COVID-19. During viral infection with SARS-CoV-2, the production of specific antibodies against the virus is consistent in most patients, except for immunodeficient patients. IgM can be found as early as 3 days after infection and provides the first line of antibodies in immunity defense, after which high-affinity IgG responses are initiated and play a key role in long-term immune memory [26]. In this study, the difference in IgG antibody levels between asymptomatic, pauci-symptomatic, and symptomatic cases achieved that the IgG levels are not significantly related with the appearance of clinical symptoms with a p-value of 0.4 >0.05. However, this study provides evidence that IgM can be positive after more than 20 days since the detection date and after symptoms have passed.

### Conclusion

In conclusion, it is shown that antigen-dependent rapid tests can be used to detect SARS CoV-2 in an early stage of infection with high sensitivity. Moreover, this study shows the age groups 21–30 and 31–40 years old have better response and are more susceptible to SARS-CoV-2 infection, and this could be because they are young and have high daily activity, most people in this age being workers. In addition, the patients’ age was significantly related to clinical symptoms. From the results of this study, we conclude that all patients who were infected with SARS-COV-2 could develop IgG and IgM antibodies after SARS-CoV-2 infection. Also, 20–30 days are not enough to give the real IgG antibody level and need longer to develop. On the other hand, the IgM level was highly related to the clinical symptoms.

### Acknowledgments

#### Conflict of interest

The authors declare that there is no conflict of interest. 

#### Ethics approval

This study obtained ethics approval from the ethical committee of the Al-Kafeel Specialized Hospital, University of Al-Ameed (UAM/EC/21-20-2021).

#### Consent to participate

All patients received informed consent.

#### Personal thanks

The authors would like to thank the department staff who supported this work.

#### Authorship

JSSA-I and ASA-S contributed to conceptualizing the study, methodology, manuscript writing, data collection, and data analysis. v
